# Predictive Value of Subacute Heart Rate Variability for Determining Outcome Following Adolescent Concussion

**DOI:** 10.3390/jcm10010161

**Published:** 2021-01-05

**Authors:** Colt A. Coffman, Jacob J. M. Kay, Kat M. Saba, Adam T. Harrison, Jeffrey P. Holloway, Michael F. LaFountaine, Robert Davis Moore

**Affiliations:** 1 Concussion Health and Neuroscience Lab, Arnold School of Public Health, University of South Carolina, Columbia, SC 29208, USA; kay2@email.sc.edu (J.J.M.K.); ksaba@email.sc.edu (K.M.S.); harri735@email.sc.edu (A.T.H.); 2 Department of Pediatrics, School of Medicine, University of South Carolina, Columbia, SC 29209, USA; Jeffrey.Holloway@uscmed.sc.edu; 3 Department of Physical Therapy, School of Health and Medical Sciences, Seton Hall University, South Orange, NJ 07110, USA; lafounmi@shu.edu; 4 Departments of Medical Sciences and Neurology, Hackensack Meridian School of Medicine, Nutley, NJ 07110, USA

**Keywords:** concussion, pediatrics, heart rate variability, adolescence, depressive symptoms, neurobehavioral function, cognition

## Abstract

Objective assessments of concussion recovery are crucial for facilitating effective clinical management. However, predictive tools for determining adolescent concussion outcomes are currently limited. Research suggests that heart rate variability (HRV) represents an indirect and objective marker of central and peripheral nervous system integration. Therefore, it may effectively identify underlying deficits and reliably predict the symptomology following concussion. Thus, the present study sought to evaluate the relationship between HRV and adolescent concussion outcomes. Furthermore, we sought to examine its predictive value for assessing outcomes. Fifty-five concussed adolescents (12–17 years old) recruited from a local sports medicine clinic were assessed during the initial subacute evaluation (within 15 days postinjury) and instructed to follow up for a post-acute evaluation. Self-reported clinical and depressive symptoms, neurobehavioral function, and cognitive performance were collected at each timepoint. Short-term HRV metrics via photoplethysmography were obtained under resting conditions and physiological stress. Regression analyses demonstrated significant associations between HRV metrics, clinical symptoms, neurobehavioral function, and cognitive performance at the subacute evaluation. Importantly, the analyses illustrated that subacute HRV metrics significantly predicted diminished post-acute neurobehavioral function and cognitive performance. These findings indicate that subacute HRV metrics may serve as a viable predictive biomarker for identifying underlying neurological dysfunction following concussion and predict late cognitive outcomes.

## 1. Introduction

The clinical management of adolescent concussion has generally relied upon subjective symptom reporting and neuropsychological assessments for determining the recovery status [1,2,3]. Despite the frequent utilization of these clinical assessments, their validity and reliability have been challenged [4]. Evidence suggests that young adults and adolescents may intentionally minimize self-reported symptom severity [5,6,7,8]. Post-concussion symptoms appear to be nonspecific, making it difficult for clinicians to identify latent deficits in function that can extend beyond the typical window of recovery, which is approximately four weeks postinjury [9,10]. Between 2001–2012, the adolescent age bracket (10–19 years old) was found to have the greatest increase in concussion incidence rate (>140%) compared to any other group [11]. The period of adolescence marks a critical stage in neurological development, and as such, the underlying maturation process in adolescents may contribute to the higher reported susceptibility to persistent deficits compared to their adult counterparts [12,13]. The shortcomings in clinical assessment strategies to formulate recovery prognoses raise concern, as premature return to a sport or academics may expose concussed adolescents to a greater risk of reinjury [14] or symptom re-emergence [15]. Thus, more objective markers of the outcome are essential for implementing an appropriate management plan for concussed adolescents.

Heart rate variability (HRV) is a widely used and reliable index of cardio-autonomic function [16], which has recently emerged as a potential biomarker for monitoring concussion outcomes [17]. In a resting state, HRV quantifies the intrinsic beat-to-beat variations in heart rate that arise through the Vagus nerve to reflect the inhibitory actions of parasympathetic nervous system function on the sinoatrial node [18,19]. The Neurovisceral Integration model proposes an important functional relationship between HRV and the regulation of cognitive and behavioral processes [20]. Multiple studies indicate HRV can peripherally index top-down activity from the prefrontal neural structures, as HRV modulates as a function of changes in prefrontal activation, as well as behavioral functioning that preferentially activates the prefrontal cortex [21,22]. In healthy individuals, greater HRV indicates that the autonomic nervous system (ANS) has sufficient reactivity to regulate physiological systems at rest [23,24]. In response to physiological stressors, healthy individuals display a relative degree of vagal withdrawal in order to meet neurometabolic demands, which is followed by a subsequent reduction in HRV [25,26]. Deviations in HRV at rest or in response to stress may indicate that dysfunction is present and may result from neurological disorders such as a traumatic brain injury (TBI) [27,28]. Preliminary research shows that concussed individuals may demonstrate exaggerated and/or blunted cardio-autonomic activity and reactivity (i.e., abnormal HRV) compared to uninjured controls [29,30,31,32,33]. Therefore, alterations to HRV that occur in concussed individuals may also indicate the presence of cognitive and neurobehavioral deficits that could otherwise go undetected with standard clinical assessment approaches, especially in adolescent cases.

Recent evidence has shown that HRV metrics are associated with symptom severity and postinjury deficits in cognitive function following a concussion [34,35]. Although promising, the current literature is incomplete and has not yet examined HRV as a predictive marker of the adolescent concussion outcome. Accordingly, our objectives were to (1) examine the associations between subacute resting-state HRV at rest and during a brief physiological stressor and adolescent concussion outcome by evaluating the clinical and depressive symptoms, neurobehavioral function, and cognitive performance and (2) investigate the predictive value of subacute HRV for determining a post-acute outcome. As such, we hypothesized that (1) HRV metrics would be associated with post-concussion outcomes at the subacute evaluation, and (2) subacute HRV metrics would predict a post-acute concussion outcome.

## 2. Experimental Section

### 2.1. Procedure

This study is a retrospective analysis of data extracted from part of a larger study on the clinical evaluation of concussions. Four-hundred and twelve youths suspected of having recently sustained a concussion were evaluated at a local pediatric sports medicine clinic. During the initial evaluation, the concussion diagnoses were confirmed by the attending physician affiliated with this study (JPH) using guidelines established by the Consensus Statement on Concussion in Sport [1] and the American Academy of Neurology [36]. Concussed participants were instructed to return for a subsequent follow-up evaluation approximately 3 weeks following the initial evaluation. All participant data were deidentified prior to data collection and analyses. Study procedures were approved by the Health Sciences South Carolina Institutional Ethics Review Board (Reference #: Pro00075286). Written consent was waived as our comprehensive concussion evaluation subserves the standard of clinical care.

### 2.2. Participants

**Figure 1:**
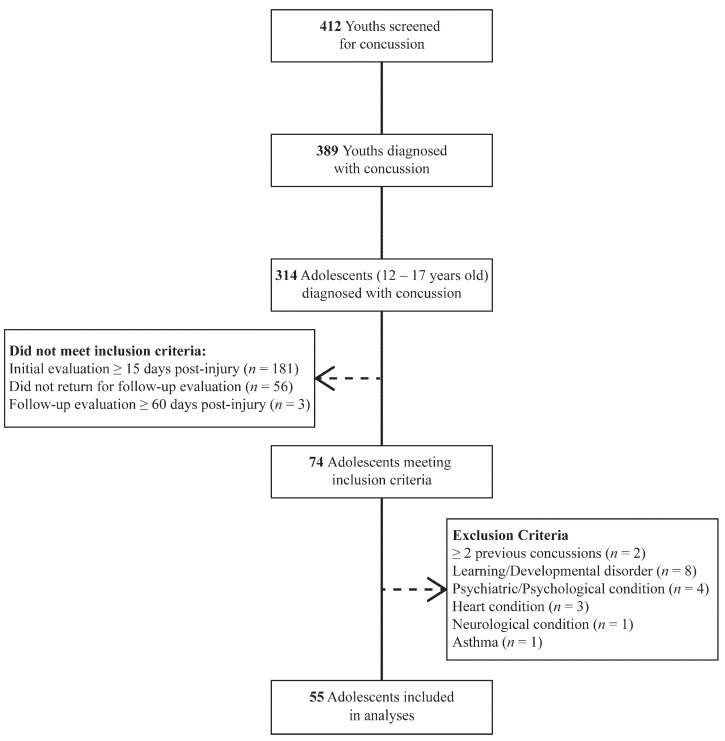
Flow diagram of the sample participants.

Of the youth screened for concussion, 74 (~18%) adolescent patients that initially received a diagnosis of concussion during subacute evaluation (3–15 days) and returned for a follow-up post-acute evaluation within 60 days of injury were identified for further analysis. Exclusion criteria included medical histories known to further alter the outcome following concussion and cardio-autonomic function. Fifty-five (13.3%) patients were eligible and included in the final analyses per the inclusion/exclusion criteria (Figure 1).

### 2.3. Measures

#### 2.3.1. Demographics/Health Information Survey

Comprehensive demographic information (e.g., age, sex, and ethnicity); injury characteristics; and medical and sport histories were collected from parents/legal guardians that accompanied the patients. Information was used to screen for possible inclusion/exclusion criteria and potential moderating variables following adolescent concussion, such as a prior diagnosis of concussion, body mass index (BMI), time since injury, and athletic status.

#### 2.3.2. Heart Rate Variability (HRV)

HRV metrics were collected through an EmWave Pro Plus infrared pulse plethysmograph ear sensor (HeartMath, Boulder Creek, CA, USA). Participants maintained a respiratory rate of 0.13 Hz (i.e., 7.5 breaths/min) in a seated position for the duration of a 5-min recording. HRV measurements occurred in a temperature and light-controlled environment at approximately the same time of day across subjects. Raw HRV data were processed using Kubios HRV Standard version 3.0.2 (Biosignal Analysis and Medical Imaging Group, Kuopio, Finland). HRV data were inspected and corrected for artifacts. Once artifacts were removed, a 10% Hanning window was applied to the corrected data. Finally, time domain and nonlinear HRV metrics were calculated. Data collection and analysis were performed in accordance with the recommendations by the Task for Force of the European Society of Cardiology and North American Society of Pacing and Electrophysiology [37].

Time domain parameters were calculated from the intervals between adjacent QRS complexes resulting from sinus node depolarization, also known as RR intervals [37]. Normalized RR intervals, free of ectopic beats and artifacts, are referred to as normal-to-normal (NN) intervals. Time domain parameters included the average difference between the highest and lowest heart rates during each respiratory cycle (HR dispersion), standard deviation of NN intervals (SDNN), and root mean square of successive NN interval differences (RMSSD) [24,38]. SDNN is known to reflect the total cardiac variability, while RMSSD solely reflects the vagal tone [39]. HR dispersion reflects respiratory sinus arrhythmia (RSA), the normal variation in heart rate that occurs during the respiration cycle independent of vagal tone [24]

Nonlinear indices have also been shown to be affected following concussion [40]. Thus, sample entropy (SampEn) was obtained using an autoregressive model to provide a more reliable measure of irregularity or complexity within a time series [24,38]. Low values of SampEn indicate a more predictable signal (i.e., lower HRV).

Additionally, a 1-min HRV recording during physical exertion (isometric handgrip contraction; IHGC) was conducted to serve as a quantifiable index of one’s ability to adapt to environmental stress. Nonlinear indices have not been validated for ultra-short-term recordings (<5 min); thus, only time domain metrics were calculated [41].

#### 2.3.3. Clinical Symptoms

Clinical symptoms were measured using the Rivermead Post-Concussion Symptoms Questionnaire (RPQ), a 16-item self-report questionnaire used to examine the severity of post-concussion symptoms compared to premorbid levels [42]. A reliable three-factor model was used to evaluate the somatic (e.g., headache, nausea, and dizziness); emotional (e.g., irritability, frustration, and restlessness); and cognitive (e.g., forgetfulness, poor concentration, and taking longer to think) symptom domains [43]. Higher RPQ subdomain scores reflect greater symptom severity.

#### 2.3.4. Depressive Symptoms

Depressive symptoms were evaluated using the Beck Youth Inventory Second Edition–Depression Scale (BYI-2), a 20-item self-report questionnaire [44]. BYI-2 questions consist of items such as sadness, pessimism, guilt, loss of pleasure, and fatigue. The BYI-2 has good test–retest reliability (0.74–0.93) and convergent validity with other instruments used to assess depressive symptoms in youths [44]. Higher total scores indicate more severe depressive symptoms.

#### 2.3.5. Neurobehavioral Function

Neurobehavioral function was assessed using the Behavior Rating Inventory of Executive Function (BRIEF-P), an 86-item parent-reported inventory of their child’s everyday executive function in the home and school environments [45]. This scale has good test–retest reliability (0.72–0.84) and internal consistency (0.80–0.98) [45]. The BRIEF-P includes two broad indices measuring various subdomains of executive function: the Behavioral Regulation Index (e.g., Inhibit, Shift, and Emotional Control) and the Metacognition Index (e.g., Initiate, Working Memory, Plan/Organize, Organization of Materials, and Monitor). Higher scores on each scale indicate greater neurobehavioral impairment.

#### 2.3.6. Cognitive Performance

Cognitive performance was tested using a modified CogState Brain Injury Testing Battery (CogState Ltd., Melbourne, Australia) consisting of three computerized task conditions. The three selected tasks included Groton Maze Learning (GML), One-back (ONB), and Groton Maze Delayed-Recall (GMR) tasks. Correct moves per second and total errors were used to assess cognitive efficiency and working memory during the Groton maze tasks. Accuracy (proportion correct), mean reaction time (msec), and reaction time variability (standard deviation of performance speed) were used to assess working memory and attention during the ONB task. Cognitive tasks incorporated into this modified battery have shown acceptable validity and good reliability across various age-groups and clinical populations [46,47,48]. In order to minimize practice effects associated with repeated assessment, practice tests were administered prior to each task [49].

### 2.4. Data Analysis

All statistical analyses were conducted using SPSS software version 27.0 (IBM Corporation, Armonk, NY, USA). To account for skewed distributions of dependent variables, the RPQ, BYI-2, and BRIEF-P scores were transformed using a natural-logarithm transformation. Additionally, ONB accuracy was transformed using an arcsine square root of accuracy, and ONB mean reaction time was transformed using a logarithmic transformation of the mean reaction time in order to enhance the inference of models. Paired-sample *t*-tests were used to examine differences in outcome measures across the subacute and post-acute evaluations. Multivariate linear regression was used to analyze (1) the association between HRV metrics and concussion outcomes at the subacute evaluation and (2) the predictive value of subacute HRV metrics for determining post-acute outcomes. Covariates known to influence HRV and post-concussion outcomes were entered into regression models, including age, sex, history of concussion, body mass index (BMI), time since injury, and athletic status (athlete/nonathlete). All models met the assumptions of linearity, constant error variance, and the absence of significant outliers and multicollinearity. A priori level of statistical significance was set to *p* < 0.05.

## 3. Results

### 3.1. Participant Characteristics

The participant demographic information and injury characteristics are summarized in Table 1. The descriptive HRV values (mean, SD, and percentiles) during rest and IHGC in the subacute evaluation can be found in Table 2.

Table 3 presents the means and standard deviations for the outcome variables at the subacute and post-acute evaluations. Paired-sample *t*-tests found that the clinical and depressive symptoms significantly decreased from the subacute to post-acute evaluation (*p* < 0.001). Measures of the neurobehavioral functions did not significantly change over time, with the exception of the Organization of Materials scale (Cohen’s *d* = 0.313, *p* = 0.024). Measures of the cognitive performances significantly improved from the subacute to post-acute evaluation (*p* < 0.05), excluding the GMR total errors and ONB reaction time (RT) variability.

### 3.2. Clinical Symptoms

The multivariate regression analyses revealed that the subacute somatic symptoms had a significant negative association with SDNN (β = -0.308, sR^2^ = 0.082) and RMSSD (β = −0.293, sR^2^ = 0.072), whereby concussed adolescents with greater HRV at rest reported lesser somatic symptom severity at the subacute evaluation. HR dispersion at rest was found to have a significant positive association with emotional (β = 0.355, sR^2^ = 0.104) and cognitive (β = 0.341, sR^2^ = 0.097) symptoms at the subacute evaluation. Subacute HRV metrics were not found to significantly predict the self-reported symptom severity on the RPQ at the post-acute evaluation (Table 4).

### 3.3. Depressive Symptoms

The subacute HRV metrics were not associated with the BYI-2 scores at either the subacute or post-acute evaluations (Table 5).

### 3.4. Neurobehavioral Function

The multivariate regression analyses found that poorer subacute Behavioral Regulation Index scores of the BRIEF-P were significantly associated with a greater resting-state SDNN (β = 0.334, sR^2^ = 0.096) and RMSSD (β = 0.303, sR^2^ = 0.078) at the subacute evaluation. In contrast, a significant negative association was found between the SampEn at rest and Behavioral Regulation Index scores at the subacute evaluation (β = −0.376, sR^2^ = 0.114), whereby a lesser SampEn was associated with poorer Behavioral Regulation scores of the BRIEF-P. No subacute HRV metrics were significantly associated with the subacute Metacognition Index scores of the BRIEF-P (Table 6).

**Figure 2:**
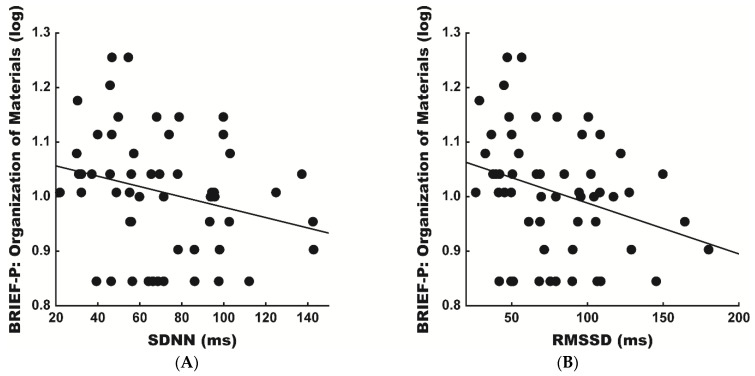
Scatter plots of the unadjusted relationships in the post-acute Behavior Rating Inventory of Executive Function (BRIEF-P) Organization of Materials scales and subacute heart rate variability (HRV) metrics at rest: (**A**) standard deviation of normal-to-normal (NN) intervals (SDNN; β = −0.255, sR^2^ = 0.065, *p* = 0.061) and (**B**) root mean square of successive NN interval differences (RMSSD; β = −0.302, sR^2^ = 0.091, *p* = 0.025). Adjusted subacute SDNN (β = −0.301, sR^2^ = 0.078, *p* = 0.043) and RMSSD (β = −0.345, sR^2^ = 0.101, *p* = 0.021) significantly predicted the post-acute BRIEF-P Organization of Materials scales.

The post-hoc analyses across the more specific executive function scales of the BRIEF-P found that the scores of the Organization of Materials scale were significantly predicted by both the subacute SDNN (β = −0.301, sR^2^ = 0.078) and RMSSD (β = −0.345, sR^2^ = 0.101) at rest, whereby the lesser SDNN and RMSSD values were associated with poorer Organization of Materials scores at the post-acute evaluation (Figure 2). The Organization of Materials scale includes questions that assess the child’s ability to establish and maintain meaningful order of their external environment [45]. No additional BRIEF-P executive function scales were significantly predicted by the subacute HRV metrics.

### 3.5. Cognitive Performance

Regarding cognitive tasks, the multivariate regression analyses revealed that lower correct moves per second on GML were significantly associated with greater SDNN (β = −0.318, sR^2^ = 0.087) and RMSSD (β = −0.346, sR^2^ = 0.101) at rest during the subacute evaluation (Table 7). HR dispersion at rest was significantly associated with the correct moves per second on GMR (β = −0.318, sR^2^ = 0.084), GMR total errors (β = 0.496, sR^2^ = 0.204; Table 8), ONB reaction time variability (β = 0.495, sR^2^ = 0.203), and ONB accuracy (β = −0.298, sR^2^ = 0.074; Table 9) at the subacute evaluation. Furthermore, greater RMSSD during IHGC was associated with a slower reaction time (β = 0.241, sR^2^ = 0.054) and greater reaction variability (β = 0.343, sR^2^ = 0.108) on the ONB task (Table 9). During IHGC, HR dispersion was associated with the correct moves per second on GML (β = −0.278, sR^2^ = 0.074; Table 7) and reaction time variability on the ONB task (β = 0.402, sR^2^ = 0.154; Table 9) at the subacute evaluation. A greater SampEn was associated with better ONB accuracy (β = 0.313, sR^2^ = 0.079; Table 9).

Additionally, SampEn significantly predicted the correct moves per second on GML (β = 0.298, sR^2^ = 0.070) at the post-acute evaluation (Table 7). Analogous to the subacute evaluation, a greater HR dispersion at rest significantly predicted a poorer cognitive performance measured by the GMR total errors (β = 0.316, sR^2^ = 0.085; Table 8). Furthermore, HR dispersion at rest (β = −0.373, sR^2^ = 0.118) and during IHGC (β = −0.319, sR^2^ = 0.097) significantly predicted ONB accuracy at the post-acute evaluation (Table 9). SDNN (β = 0.278, sR^2^ = 0.070) and RMSSD (β = 0.328, sR^2^ = 0.099) during IHGC significantly predicted the ONB reaction time at the post-acute evaluation (Table 9).

## 4. Discussion

The purpose of the present study was to investigate the association and value of HRV during rest and physical exertion to predict concussion outcomes in adolescent patients. We found that HRV metrics were associated with clinical symptoms and neurobehavioral function at the subacute evaluation but did not predict the post-acute symptom severity. Furthermore, the HRV indices quantified during this investigation were neither associated with nor predicted depressive symptoms. However, HRV metrics at rest and during a physical exertion task did predict the neurobehavioral regulation and cognitive performance at the post-acute evaluation, suggesting it may be of clinical utility for healthcare providers.

Following concussion, the autonomic nervous system (ANS) and cardiovascular system decouple on multiple levels [50]. This form of autonomic dysregulation includes sympathetic hyperarousal (e.g., decreased regulation of norepinephrine) and altered hypothalamic–pituitary axis functioning (e.g., increased cortisol). In addition, glucometabolic decoupling in the brain leads to a marked increase in neurometabolic demand following concussion [51]. The Neurovisceral Integration Model states that HRV serves as a means of quantifying the efficiency of neural communication between higher-order prefrontal structures and physio-regulatory systems (i.e., cardiovascular) [16,38]. The authors suggest that dynamic connectivity between such regions is necessary to coordinate behavioral responses to meet the metabolic demand [52]. Concussion produces a transient loss of functional connectivity in the brain [53,54,55], which perpetuates cardio-autonomic impairment. Cardio-autonomic impairment is commonly reflected by abnormal HRV following a concussive brain injury.

Accordingly, we observed that diminished SDNN and RMSSD were significantly associated with more severe somatic symptomology at the subacute evaluation. Our findings are consistent with observations of attenuated resting-state HRV during the acute and subacute recovery phases, often characterized by the presence of concussion symptoms [29,30]. It is hypothesized that the ability to acutely depress the basal metabolic rate following concussion is necessary to foster neuronal rehabilitation [51,56]. At rest, the ANS actively engages a vagal “brake” on sympathetic activity to protect the oxygen-dependent central nervous system from costly metabolic reactions [57]. Therefore, the relationship observed between a greater HRV and lesser severe symptoms could reflect that an early hypometabolic resting state is needed to support recovery following concussive brain injury. However, during the post-acute phase of injury, concussed and non-injured adolescents rarely differ in measures of cardio-autonomic function at rest [28,58,59]. This evidence may explain why associations between subacute HRV and post-acute clinical symptoms were not present in the current study.

In contrast, we did not observe a relationship between the metrics of HRV and depression at either timepoint. This finding was contrary to our hypotheses and prior evidence suggesting that HRV metrics may predict a later onset of depressive symptoms in adult females with a mTBI (mild traumatic brain injury) [60]. However, prior research examined adults and used the adult version of the Beck Depression Inventory, which contains related but different questions [61]. It is also worth noting that the menstrual cycle phase at the time of injury has been shown to impact the postinjury symptoms and quality of life at one-month postinjury [62]. Given the ages of our participants and statistical analyses combining both sexes into the models, the possibility exists that differences within our female participants in the context of menarche, eumenorrhea, and the use of hormone-based contraceptives could have contributed to our lack of similar observations. With further data collections and a larger sample size of females, the analyses may elucidate potential sex-based differences.

In addition, we observed that higher indices of vagal activity were associated with poorer behavioral regulation scores (SDNN and RMSSD) and slower cognitive performance as indexed by correct moves per second on GML (SDNN and RMSSD) at the subacute evaluation. Furthermore, a greater HRV during physical exertion was associated with slower cognitive performance as indexed by the reaction time and reaction time variability on the ONB task at the subacute evaluation (RMSSD). These findings corroborate prior research that observed associations between higher HRV and poorer emotional and cognitive symptoms in concussed adolescents [35]. Recent literature demonstrates that concussed patients may exhibit an inappropriate increase in vagal tone in response to physiological and cognitive stress compared to healthy controls [32,33]. The current theories propose that a failure to suppress the vagal tone may reflect an inability to orient one’s resources toward environmental stressors [63]. As such, we further observed that greater SDNN and RMSSD during physical exertion predicted a slower cognitive performance on the ONB task at the post-acute evaluation. With the existing literature in mind, these findings may indicate that acutely concussed adolescents with higher HRV may exhibit an inability to adequately withdraw vagal resources in response to stressors. Interestingly, we observed that higher subacute HRV at rest predicted better “external” organization (SDNN and RMSSD) scores at the post-acute evaluation. These contradicting results are perplexing and warrant further investigation into the relationship between HRV at rest and during physical challenges and organizational skills in adolescents.

Beyond traditional HRV variables, we observed consistent associations between HR dispersion and concussion outcomes across the timepoints. We observed that those with greater HR dispersion reported more severe emotional and cognitive symptoms, as well as performed worse on cognitive tasks at the subacute evaluation. Moreover, a greater HR dispersion at rest and during stress predicted a poorer cognitive performance on the GMR and ONB tasks at the post-acute evaluation. These findings corroborate those of Brandt and colleagues, who identified RSA indices as potential predictors of adverse mTBI outcomes [64].

The preliminary research also suggests that HRV complexity (i.e., ApEn; approximate entropy/SampEn) may be attenuated during the acute, subacute, and post-acute phases of concussion recovery [40,65]. Accordingly, we observed that lower SampEn values were significantly associated with poorer neurobehavioral regulation scores and ONB accuracy at the subacute evaluation. Lower subacute SampEn also predicted slower GML performance at the post-acute evaluation, suggesting adolescents with attenuated HRV complexity exhibit persisting neurobehavioral and cognitive dysfunctions. Although, both HR dispersion and SampEn present beneficial values in predicting post-concussion outcomes, their true values as physiological biomarkers for clinicians has yet to be determined.

Together, our results help advance the scientific understanding of the relations between HRV and concussion outcomes. Our results are the first to suggest that HRV may predict neurobehavioral and cognitive outcomes beyond the subacute phase of injury. This is an important point, as prior subacute associations between HRV across domains of functioning have led many researchers and clinicians to suggest that HRV is just a general marker of dysfunction bereft of specificity and predictive power. However, our results point to a specific predictive value of HRV to post-acute neurobehavioral functions and cognitive functions independent of symptom questionnaires. Thus, HRV may be a simple and effective way for clinicians to identify patients who are likely to experience persisting alterations in neurobehavioral functioning and cognition. This would provide clinicians the opportunity to preemptively change management strategies to maximize the positive outcomes.

Although the current results serve to provide a specific relation between HRV and concussion outcomes, the present study is not without limitations. First, although age was adjusted for in our regression analyses, our results may not fully account for individual differences in physiological maturity among this sample of adolescents. The current findings also cannot inform us regarding the differences in female populations across estrous cycles or across birth control types. Although both sexes were represented in the current study, menarche, estrous phase, and hormonal contraceptives can influence HRV. Furthermore, as this study was conducted in a clinic and not a laboratory, we cannot determine the central mechanisms (biochemical and psychophysiological) that mediate the relation between HRV metrics and functional outcomes. Additionally, as the preinjury baseline measures were not captured, the current study cannot tell us how these relations may change as a function of sport and sub-concussive impacts, which affect the neuronal integrity centrally and peripherally. Furthermore, while we controlled for the BMI and physical activity level, we did not have objective measures of cardiorespiratory fitness, which may influence the resting HR and HRV. Thus, additional research is still needed from a scientific and clinical perspective to determine the predictive value of HRV across different populations and demographic factors.

## 5. Conclusions

The present study is the first to suggest that acute HRV at rest and during a physical exertion task may be useful for predicting neurobehavioral and cognitive outcomes in the post-acute phase of injury. Importantly, our results fit with the prevailing model of cardio-autonomic regulation, which posits a strong relation between HRV, learning, memory, and attention. Thus, our findings provide both a theoretical and data-driven impetus for using HRV in the clinical assessment and management of concussions. HRV is feasible for use in clinical settings, as it is noninvasive; cost-effective; and can be used under various conditions (e.g., rest, exercise, and cognition). Furthermore, measuring the HRV does not require the level of training or space of other psychophysiological measures and can be collected from simple ear clips or three-lead ECGs (Electrocardiogram). That being said, further research is needed to understand the relation of HRV to concussion outcomes across a wide variety of populations and demographic factors in order to provide clinicians with a more complete understanding of the benefits and limitations of HRV to their clinical practice.

## Figures and Tables

**Table 1:** Participant demographic information and injury characteristics.

	Participant Data (*n* = 55)
**Demographic Information**	
Age (years)	14.5 ± 1.4
BMI (kg/m^2^)	24.4 ± 6.1
Biological Sex, N (%)	
Males	31 (56.4)
Females	24 (43.6)
Ethnicity, N (%)	
Caucasian	22 (40)
African American	21 (38.2)
Latino/Hispanic	2 (3.6)
Native American	1 (1.8)
Other/Unknown	9 (16.4)
History of Concussion, N (%)	
No History	43 (78.2)
One Prior Concussion	12 (21.8)
Athlete Status, N (%)	
Athlete	41 (74.5)
Nonathlete	14 (25.5)
**Injury Characteristics**	
Cause of Injury, N (%)	
Sport or Recreation	37 (67.3)
Motor Vehicle Accident	11 (20.0)
Other (fall, accident, etc.)	7 (12.7)
Days from Concussion	
Subacute Evaluation (3–15 days)	9.0 ± 4.5
Post-Acute Evaluation (15–60 days)	29.2 ± 10.2

Note: Data are reported as mean ± SD, unless otherwise noted. BMI: body mass index.

**Table 2:** Descriptive HRV values for concussed participants at the subacute evaluation.

HRV Variable	Mean ± SD	25th Percentile	50th Percentile	75th Percentile
Resting State				
HR dispersion	28.8 ± 8.8	25.0	28.0	32.4
SDNN	71.4 ± 29.7	47.8	68.0	94.3
RMSSD	79.2 ± 35.9	49.7	71.5	103.3
SampEn	1.75 ± 0.21	1.68	1.76	1.90
Isometric Handgrip Contraction				
HR dispersion	29.8 ± 1.3	21.7	29.6	35.7
SDNN	114.4 ± 6.9	83.3	106.1	143.7
RMSSD	93.9 ± 8.1	53.0	75.5	111.8

Abbreviations: HRV, heart rate variability; HR, heart rate; SDNN, standard deviation of normal-to-normal (NN) intervals; RMSSD, root mean square of successive NN interval differences; and SampEn, sample entropy.

**Table 3:** Descriptive outcome values for concussed participants at the subacute and post-acute evaluations.

Outcome Variable		Mean ± SD	Cohen’s *d*	*p*-Value
**RPQ subdomain**				
Somatic	Subacute	11.0 ± 6.9	0.931	<0.0001 *
	Post-Acute	5.6 ± 6.9		
Emotional	Subacute	4.3 ± 3.6	0.985	<0.0001 *
	Post-Acute	2.0 ± 3.2		
Cognitive	Subacute	4.9 ± 3.2	1.067	<0.0001 *
	Post-Acute	2.0 ± 2.7		
**BYI-2 Depression Scale**				
Total Score	Subacute	0.67 ± 0.44	0.637	<0.0001 *
	Post-Acute	0.41 ± 0.47		
**BRIEF-P subdomain**				
Behavioral Regulation Index	Subacute	36.9 ± 8.5	0.112	0.412
	Post-Acute	36.5 ± 10.3		
Metacognition Index	Subacute	66.5 ± 17.3	0.247	0.073
	Post-Acute	63.8 ± 17.0		
**CogState**				
GML correct moves per second	Subacute	0.60 ± 0.16	0.884	<0.0001 *
	Post-Acute	0.73 ± 0.17		
GML total errors	Subacute	59.4 ± 16.9	0.586	<0.0001 *
	Post-Acute	50.8 ± 16.5		
GMR correct moves per second	Subacute	0.85 ± 0.24	0.520	<0.0001 *
	Post-Acute	0.97 ± 0.30		
GMR total errors	Subacute	7.6 ± 4.4	0.165	0.225
	Post-Acute	6.8 ± 4.4		
ONB reaction time (ms)	Subacute	1071.2 ± 319.2	0.307	0.027 *
	Post-Acute	990.0 ± 269.5		
ONB RT variability	Subacute	0.15 ± 0.03	0.102	0.451
	Post-Acute	0.14 ± 0.04		
ONB accuracy (%)	Subacute	84.5 ± 15.4	0.308	0.026 *
	Post-Acute	89.0 ± 0.10		

Note: Cohen’s *d* and *p*-values were derived from paired-sample *t*-tests. * denotes statistical significance (*p* < 0.05). RPQ, Rivermead Post-Concussion Symptoms Questionnaire; BYI-2, Abbreviations: Beck Youth Inventory-2; BRIEF-P, Behavior Rating Inventory of Executive Function; GML, Groton Maze Learning; GMR, Groton Maze Recall; and ONB RT, One-back task reaction time.

**Table 4:** Multivariate regression analyses for the Rivermead Post-Concussion Symptom Questionnaire (RPQ) ^a^.

	Subacute RPQ Symptom Domain								
	Somatic			Emotional			Cognitive		
HRV Variable	β	sR^2^	*p*	β	sR^2^	*p*	β	sR^2^	*p*
Resting State									
HR dispersion	0.180	0.027	0.200	0.355	0.104	0.009 *	0.341	0.097	0.016 *
SDNN	−0.308	0.082	0.023 *	0.101	0.009	0.463	−0.024	0.000	0.870
RMSSD	−0.293	0.072	0.033 *	0.114	0.011	0.413	−0.042	0.001	0.774
SampEn	0.169	0.023	0.238	−0.180	0.026	0.205	−0.081	0.005	0.584
Isometric Handgrip Contraction									
HR dispersion	0.012	0.000	0.929	0.240	0.055	0.064	−0.161	0.025	0.232
SDNN	−0.163	0.024	0.226	0.154	0.021	0.253	−0.120	0.013	0.391
RMSSD	−0.047	0.002	0.726	0.212	0.042	0.109	−0.077	0.005	0.576
	**Post-Acute RPQ Symptom Domain**								
Resting State									
HR dispersion	−0.002	0.000	0.986	0.046	0.002	0.735	0.124	0.013	0.380
SDNN	0.024	0.001	0.851	0.001	0.000	0.993	−0.009	0.000	0.948
RMSSD	0.073	0.005	0.573	0.066	0.004	0.627	0.033	0.001	0.815
SampEn	−0.047	0.001	0.725	−0.018	0.000	0.898	0.034	0.001	0.820
Isometric Handgrip Contraction									
HR dispersion	0.007	0.000	0.954	−0.028	0.001	0.826	−0.045	0.002	0.736
SDNN	0.026	0.001	0.837	−0.003	0.000	0.981	−0.053	0.003	0.701
RMSSD	0.033	0.001	0.794	−0.013	0.000	0.920	−0.009	0.000	0.945

^a^ Age, sex, history of concussion, BMI, time since injury, and athletic status were entered into the adjusted models. * denotes predictor significance (*p* < 0.05). sR^2^, squared semi-partial correlations. Abbreviations: HRV, heart rate variability; HR, heart rate; SDNN, standard deviation of NN intervals; RMSSD, root mean square of successive NN interval differences; and SampEn, sample entropy.

**Table 5:** Multivariate regression analyses for the Beck Youth Inventory–Depression Scale (BYI-2) ^a^.

	Subacute BYI-2 Scores			Post-Acute BYI-2 Scores		
HRV Variable	β	sR^2^	*p*	β	sR^2^	*p*
Resting State						
HR dispersion	−0.031	0.000	0.840	0.024	0.000	0.867
SDNN	−0.107	0.010	0.474	0.098	0.008	0.482
RMSSD	−0.073	0.004	0.632	0.132	0.015	0.345
SampEn	−0.002	0.006	0.569	−0.112	0.010	0.438
Isometric Handgrip Contraction						
HR dispersion	−0.019	0.000	0.892	0.030	0.001	0.820
SDNN	−0.055	0.003	0.706	0.051	0.002	0.709
RMSSD	0.051	0.002	0.724	0.031	0.008	0.820

^a^ Age, sex, history of concussion, BMI, time since injury, and athletic status were entered into the adjusted models. No significance was observed (*p* > 0.05). sR^2^, squared semi-partial correlations. Abbreviations: HRV, heart rate variability; HR, heart rate; SDNN, standard deviation of NN intervals; RMSSD, root mean square of successive NN interval differences; and SampEn, sample entropy.

**Table 6:** Multivariate regression analyses for the Behavior Rating Inventory of Executive Function (BRIEF-P) ^a^.

	Subacute BRIEF-P Index					
	Behavioral Regulation Index			Metacognition Index		
HRV Variable	β	sR^2^	*p*	β	sR^2^	*p*
Resting State						
HR dispersion	0.010	0.000	0.948	0.071	0.004	0.627
SDNN	0.334	0.096	0.017 *	0.102	0.009	0.476
RMSSD	0.303	0.078	0.034 *	0.080	0.005	0.580
SampEn	−0.376	0.114	0.009 *	−0.126	0.013	0.392
Isometric Handgrip Contraction						
HR dispersion	0.196	0.036	0.149	0.210	0.042	0.117
SDNN	0.133	0.016	0.345	0.019	0.000	0.893
RMSSD	0.068	0.004	0.628	−0.038	0.001	0.784
	**Post-Acute BRIEF-P Index**					
Resting State						
HR dispersion	0.058	0.003	0.691	0.122	0.013	0.424
SDNN	−0.039	0.001	0.784	−0.130	0.015	0.387
RMSSD	−0.010	0.000	0.946	−0.132	0.015	0.387
SampEn	−0.045	0.002	0.764	0.025	0.000	0.875
Isometric Handgrip Contraction						
HR dispersion	0.015	0.000	0.914	0.011	0.000	0.940
SDNN	−0.062	0.003	0.661	−0.161	0.023	0.276
RMSSD	0.001	0.000	0.995	−0.133	0.016	0.359

^a^ Age, sex, history of concussion, BMI, time since injury, and athletic status were entered into the adjusted models. * denotes predictor significance (*p* < 0.05). sR^2^, squared semi-partial correlations. Abbreviations: HRV, heart rate variability; HR, heart rate; SDNN, standard deviation of NN intervals; RMSSD, root mean square of successive NN interval differences; and SampEn, sample entropy.

**Table 7:** Multivariate regression analyses for Groton Maze Learning (GML) ^a^.

	Subacute Cognitive Performance (GML)					
	GML Correct Moves Per Second			GML Total Errors		
HRV Variable	β	sR^2^	*p*	β	sR^2^	*p*
Resting State						
HR dispersion	−0.117	0.011	0.407	0.287	0.068	0.057
SDNN	−0.318	0.087	0.019 *	0.144	0.018	0.340
RMSSD	−0.346	0.101	0.011 *	0.139	0.016	0.359
SampEn	0.196	0.031	0.170	−0.093	0.007	0.552
Isometric Handgrip Contraction						
HR dispersion	−0.278	0.074	0.031 *	0.071	0.004	0.621
SDNN	−0.224	0.045	0.095	0.011	0.000	0.940
RMSSD	−0.249	0.057	0.059	0.030	0.001	0.839
	**Post-Acute Cognitive Performance (GML)**					
Resting State						
HR dispersion	−0.006	0.000	0.964	0.147	0.019	0.274
SDNN	−0.151	0.020	0.266	−0.023	0.000	0.865
RMSSD	−0.143	0.017	0.298	−0.035	0.001	0.795
SampEn	0.298	0.070	0.032 *	−0.130	0.013	0.353
Isometric Handgrip Contraction						
HR dispersion	−0.247	0.059	0.052	0.028	0.001	0.830
SDNN	−0.167	0.023	0.208	−0.152	0.020	0.247
RMSSD	−0.093	0.008	0.480	−0.127	0.015	0.327

^a^ Age, sex, history of concussion, BMI, time since injury, and athletic status were entered into adjusted models. * denotes predictor significance (*p* < 0.05). sR^2^, squared semi-partial correlations. Abbreviations: HRV, heart rate variability; HR, heart rate; SDNN, standard deviation of NN intervals; RMSSD, root mean square of successive NN interval differences; and SampEn, sample entropy.

**Table 8:** Multivariate regression analyses for Groton Maze Recall (GMR) ^a^.

	Subacute Cognitive Performance (GMR)					
	GMR Correct Moves per Second			GMR Total Errors		
HRV Variable	β	sR^2^	*p*	β	sR^2^	*p*
Resting State						
HR dispersion	−0.318	0.084	0.030 *	0.496	0.204	0.001 *
SDNN	0.096	0.008	0.531	−0.262	0.059	0.071
RMSSD	−0.251	0.053	0.087	0.041	0.001	0.792
SampEn	0.254	0.052	0.091	−0.170	0.023	0.282
Isometric Handgrip Contraction						
HR dispersion	−0.188	0.033	0.175	0.109	0.011	0.452
SDNN	−0.186	0.031	0.193	0.069	0.004	0.645
RMSSD	−0.263	0.063	0.060	0.127	0.012	0.390
	**Post-Acute Cognitive Performance (GMR)**					
Resting State						
HR dispersion	−0.128	0.014	0.355	0.316	0.085	0.026 *
SDNN	−0.242	0.051	0.075	0.013	0.000	0.926
RMSSD	−0.268	0.061	0.050	0.037	0.001	0.798
SampEn	0.260	0.054	0.066	−0.087	0.006	0.564
Isometric Handgrip Contraction						
HR dispersion	−0.213	0.043	0.101	−0.041	0.002	0.763
SDNN	−0.256	0.077	0.054	−0.081	0.006	0.566
RMSSD	−0.230	0.049	0.081	−0.090	0.007	0.517

^a^ Age, sex, history of concussion, BMI, time since injury, and athletic status were entered into adjusted models. * denotes predictor significance (*p* < 0.05). sR^2^, squared semi-partial correlations. Abbreviations: HRV, heart rate variability; HR, heart rate; SDNN, standard deviation of NN intervals; RMSSD, root mean square of successive NN interval differences; and SampEn, sample entropy.

**Table 9:** Multivariate regression analyses for One-Back task (ONB) ^a^.

	Subacute Cognitive Performance (ONB)								
	ONB Mean RT (ms)			ONB RT Variability			ONB Accuracy (%)		
HRV Variable	β	sR^2^	*p*	β	sR^2^	*p*	β	sR^2^	*p*
Resting State									
HR dispersion	0.052	0.002	0.666	0.495	0.203	0.001 *	−0.298	0.074	0.049 *
SDNN	0.061	0.003	0.611	0.218	0.041	0.126	−0.271	0.063	0.068
RMSSD	0.077	0.005	0.523	0.185	0.029	0.201	−0.212	0.038	0.160
SampEn	−0.095	0.007	0.438	−0.122	0.012	0.412	0.313	0.079	0.041 *
Isometric Handgrip Contraction									
HR dispersion	0.090	0.008	0.424	0.402	0.154	0.002 *	−0.195	0.036	0.169
SDNN	0.150	0.020	0.194	0.252	0.057	0.069	−0.175	0.028	0.233
RMSSD	0.241	0.054	0.032 *	0.343	0.108	0.011 *	−0.174	0.028	0.229
	**Post-acute Cognitive Performance (ONB)**								
Resting State									
HR dispersion	−0.098	0.008	0.492	−0.010	0.000	0.948	−0.373	0.118	0.008 *
SDNN	0.175	0.026	0.215	0.042	0.001	0.780	−0.206	0.037	0.149
RMSSD	0.186	0.029	0.192	0.066	0.003	0.667	−0.156	0.021	0.280
SampEn	−0.278	0.062	0.055	0.040	0.001	0.800	0.119	0.011	0.427
Isometric Handgrip Contraction									
HR dispersion	0.213	0.043	0.109	0.066	0.004	0.649	−0.319	0.097	0.016 *
SDNN	0.278	0.070	0.042 *	0.037	0.001	0.804	−0.177	0.028	0.205
RMSSD	0.328	0.099	0.014 *	0.055	0.003	0.707	−0.230	0.049	0.094

^a^ Age, sex, history of concussion, BMI, time since injury, and athletic status were entered into adjusted models. * denotes predictor significance (*p* < 0.05). sR^2^, squared semi-partial correlations. Abbreviations: RT, reaction time; HRV, heart rate variability; HR, heart rate; SDNN, standard deviation of NN intervals; RMSSD, root mean square of successive NN interval differences; and SampEn, sample entropy.

## Data Availability

The datasets generated for this study are available on request to the corresponding author.
